# Osteoarticular Infection in Three Young Thoroughbred Horses Caused by a Novel Gram Negative Cocco-Bacillus

**DOI:** 10.1155/2020/9785861

**Published:** 2020-01-18

**Authors:** Bernard J. Hudson, Catherine Chicken, Anna Blishen, Kristen H. Todhunter, Angela P. Begg, Leonie Chan, Thomas Karagiannis, Benjamin Raymond, Daniel Bogema, Angus R. Adkins, Christopher B. O'Sullivan, Brendon A. O'Rourke, Piklu Roy Chowdhury, Steven P. Djordjevic, Ian G. Charles, Andrew Edgar, Katerina Mitsakos

**Affiliations:** ^1^NSW Health Pathology, Department of Microbiology & Infectious Diseases, Royal North Shore Hospital, St Leonards, Sydney, New South Wales 2065, Australia; ^2^Scone Equine Hospital, 106 Liverpool Street, Scone, New South Wales 2337, Australia; ^3^Vetnostics Laverty Pathology, 60 Waterloo Rd, Macquarie Park, Sydney, New South Wales 2113, Australia; ^4^Newcastle Equine Centre & Newcastle Equine Rehabilitation & Reproduction Centre, The Old Tote Building, Broadmeadow Racecourse, New South Wales 2292, Australia; ^5^The ithree Institute, University of Technology, 15 Broadway, Ultimo, Sydney, New South Wales 2007, Australia; ^6^Department of Primary Industry, Elizabeth Macarthur Agricultural Institute, Woodbridge Rd, Menangle, New South Wales 2568, Australia; ^7^Randwick Equine Centre, 3 Jane St, Randwick, Sydney, New South Wales 2031, Australia; ^8^Quadrum Institute Bioscience—Institute of Food Research, Norwich Research Park, Norwich NR4 7UA, UK; ^9^Newmarket Equine Hospital, Cambridge Road, Newmarket, Suffolk CB8 OFG, UK

## Abstract

We describe three cases of osteoarticular infection (OAI) in young thoroughbred horses in which the causative organism was identified by MALDI-TOF as *Kingella species*. The pattern of OAI resembled that reported with *Kingella* infection in humans. Analysis by 16S rRNA PCR enabled construction of a phylogenetic tree that placed the isolates closer to *Simonsiella* and *Alysiella species*, rather than *Kingella species*. Average nucleotide identity (ANI) comparison between the new isolate and *Kingella kingae* and *Alysiella crassa* however revealed low probability that the new isolate belonged to either of these species. This preliminary analysis suggests the organism isolated is a previously unrecognised species.

## 1. Introduction

Septic arthritis in equines has a high mortality rate (22–58%) despite treatment [1–3]. In survivors, residual damage causing persistent osteoarthritis and lameness are common complications [4, 5]. For thoroughbred horses, septic arthritis is associated with a reduced likelihood of ever starting in a race compared to controls [6]. Septic arthritis occurs following trauma, especially penetrating injuries of the joint, spread from a contiguous focus of infection, and by haematogenous seeding of microorganisms [1, 4, 7]. The latter may be from a remote site of infection, but likely occurs in many individuals via symptomatic or asymptomatic bacteraemia [1, 3, 4, 8]. Confirmation of a bacterial cause is by positive microscopy, together with culture of synovial fluid (SF) or synovial tissue [9]. Bacterial culture of SF is negative in 33–60% of Cases [1, 3, 10, 11].

Undetected bacteria may cause equine OAI but, if an infectious etiology is the cause, nonbacterial causes could also account for a percentage of those cases with negative bacterial culture and PCR results. Alternatively, a noninfective etiology may be the reason for negative studies for infective agents. We present three (3) cases of OAI in young equines wherein a bacterium, not previously described, was isolated from bone, synovial tissue and synovial fluid (SF) submitted for culture, and discuss the relevance to OAI in equines.

## 2. Cases

The details of the three cases are listed in Supplementary [Supplementary-material supplementary-material-1]. Specific additional information is presented below. For all cases, the microbiology tests and results are discussed separately and are also listed in Supplementary [Supplementary-material supplementary-material-1]. There was more than 80 km distance between the farms where Cases 1 and 3 occurred. There was over 300 km distance between the location of Case 2 and the other two farms. There was no known contact between any of the horses and personnel on any of the farms.

*Case 1* was a thoroughbred filly, aged 15 months residing in the Hunter Valley in NSW Australia, that presented in November 2012 with progressive lameness in the right foreleg of 2 weeks' duration. On arrival the horse was bright and alert, severely lame in the right foreleg and, apart from being febrile (38.9°C), had normal vital signs and no other identified focus of infection. There was swelling and obvious pain on direct palpation of the right fore shoulder region, including the bicipital bursa. Radiographic examination of the shoulder found no significant abnormality. Ultrasound examination showed a large volume of echoic masses, most likely fibrinous material, within the bicipital bursa but only a small volume of synovial fluid. The distal aspect of the intermediate tubercle of the humerus was abnormal with a loss of the normal smooth contour. Attempted aspiration of synovial fluid from the bicipital bursa was unsuccessful, but synovial fluid was successfully aspirated from the shoulder joint. Results are listed in Supplementary [Supplementary-material supplementary-material-1].

The horse underwent general anaesthesia and endoscopic visualisation of the right shoulder joint and bicipital bursa. Synovial fluid from the bursa and joint was collected and submitted for culture. Endoscopy showed normal appearance of shoulder joint synovium, but a large amount of free and adhered fibrin in the bicipital bursa which was removed and submitted for culture. There was an area (12 mm × 15 mm) at the distal aspect of the intermediate tubercle that was devoid of fibrocartilage and was soft when probed. The area was debrided to normal subchondral tissue and this tissue was also submitted for culture. Histopathology of synovial biopsy from the bicipital bursa identified a subacute largely purulent proliferative synovitis with no bacteria detected on microscopy. Antimicrobial treatment is listed in Supplementary [Supplementary-material supplementary-material-1]. At six weeks post-operatively the horse was sound at the trot. The horse subsequently raced (and was placed) as a two-year old and was, subsequently at three and fours years of age, a multiple race winner in provincial Victoria, and placed on multiple times in metropolitan Victoria. Other progeny from the mother of this horse have been multiple stakes winners.

*Case 2* was a thoroughbred filly aged 7 months, residing in the Southern Highlands district of NSW that presented in August 2013 with lameness in the left foreleg, of uncertain duration. Despite treatment for 72 hours with doxycycline, lameness progressed and the horse was referred for further evaluation and possible surgery. On arrival the horse was bright and alert, severely lame at the walk in the left foreleg, afebrile with normal vital signs and no other identified focus of infection. There was swelling and severe pain on palpation of the proximal axial aspect of the medial splint bone. Radiographic projections showed evidence of a focal area of cortical bone resorption in the palmaromedial mid-proximal diaphyseal cortex of the left third metacarpal (McIII). Bone lucency (2 mm × 14 mm in dimension) was present in the region of the intraosseous ligament attachment between the second and third metacarpals. Ultrasound showed an obvious bone defect in the superficial cortex of McIII corresponding to the lucency seen on radiographs. Blood abnormalities were mild (see Supplementary [Supplementary-material supplementary-material-1]). At surgical exploration a small necrotic detached fragment of McIII bone was elevated and removed and submitted for microbiology. Antimicrobial treatment was with amikacin beads placed in the bone defect, ceftriaxone (regional limb perfusion), procaine penicillin G, gentamicin and thence oral trimethoprim/sulphur for seven days. At six weeks post-surgery, lameness and pain had resolved. The filly barrier trialled once in country Victoria and was sold and exported out of Australia.

*Case 3* was an unweaned male thoroughbred aged three months residing in the Hunter Valley in NSW Australia that presented in December 2016 with severe lameness and an effusion of the left hind digital flexor tendon sheath. Few other clinical details are available. Plain X-rays of the left hind fetlock and pastern were normal. The tendon sheath was lavaged and a large amount of fibrin was removed arthroscopically under general anaesthesia. Synovial fluid was described as turbid, with protein and cell count being abnormal. Antimicrobial treatment was as set out in Supplementary [Supplementary-material supplementary-material-1]. On the sixth post-operative day the horse was discharged to its home farm, sound at the walk, but no other clinical details are available. The horse subsequently raced as a two-year old, placed in a barrier trial in provincial New South Wales (NSW), but was subsequently unplaced in sixteen starts in NSW and country areas of Queensland. Other progeny from the mother of this horse have been either unraced or unplaced.

## 3. Methods

Synovial fluid was available for inoculation into Oxoid Signal blood culture bottles (BCB) (Provet Vi, Sydney, NSW, Australia) at the time of collection from surgery, for Cases 1 and 3, but was unavailable for Case 2. Other samples available for culture were fibrin from lavage from Case 1 and Case 3 and debrided bone from Case 2. All collected samples, including inoculated BCBs, were delivered on the same day to the laboratory in Cases 1 and 3 within 30 minutes of collection, and within 6 hours of collection for Case 2. All samples post collection were inoculated onto Columbia sheep blood agar and chocolate agar (both bioMerieux, Australia) and into cooked meat enrichment media (CMM) as per manufacturers recommendations (Thermo Fisher Scientific, Adelaide, Australia), as soon as they reached the laboratory. Incubation conditions for cultured media were aerobic (35°C ± 2°C and 5% carbon dioxide atmosphere) and anaerobic (35°C ± 2°C and 10% carbon dioxide atmosphere). Plates were read at 36 hours then re-incubated for up to five days, checking every 24 hours for growth, except for the anaerobic plates that were checked every 48 hours for up to five days. The CMM was incubated until positive and checked daily for visual signs of turbidity, then subcultured, if turbid, in the same manner as the cultures on the initial samples. For identification, isolates were treated as members of the *Neisseriaceae* and of the HACEK group based on initial morphological features. HACEK represents a group of Gram negative bacteria (GNB) that are part of normal human flora: *Haemophilus species, Aggregatibacter actinomycetemcomitans, Cardiobacterium hominis, Eikenella corrodens*, and *Kingella species*. These organisms can be readily identified by commercial systems such as API NH and VITEK 2 (bioMérieux, Marcy-l'Etoile, France) and matrix-assisted laser desorption ionization-time of flight mass spectrometry [12–15]. The initial isolate (Isolate 1 from Case 1) failed to propagate after repeated subculture, but the Isolate 2 and Isolate 3 (from Cases 2 and 3, respectively) survived repeated subculture on supplemented Columbia horse blood agar [16] in 5% carbon dioxide atmosphere. These isolates were sent to the laboratory at St. Leonards for more extensive testing than was done in the laboratories where the organisms were isolated originally. Isolates 2 and 3 (from Cases 2 and 3) were stored at −70°C in a 15% glycerol-containing medium, whence they were retrieved for more extensive testing.

For *Isolates 2* and *3*, motility studies were performed as per published methods [17, 18]. Bacteria were emulsified in nutrient broth, allowed to grow for 2–24 hours between 15–25°C and 37°C and broth was checked at intervals (4, 8, 24 hours). A drop of the bacterial suspension was placed onto a slide covered with a coverslip and examined using a phase contrast microscope. ATCC controls used and propagated as described by the ATCC bacterial culture guide [19], and Garitty et al. [20] were: *Enterobacter cloacae, Pseudomonas aeruginosa* ATCC 27853 (positive); *Klebsiella pneumoniae* ATCC 13882 (negative); and *Kingella kingae* ATCC 23330 (twitching motility as occurs with the extension and retraction of the type IV pilus).

Biochemical tests were performed on subcultures after 36 hours of incubation on Columbia horse blood agar (Oxoid) [16]. Limited tests were done on Isolate 1, as it did not survive repeated subculture. For Isolates 2 and 3 the following were utilised: ID32 STAPH version 3, API STAPH version 5, rapid ID 32 STREP version 4, API 20 STREP, version 8, API 20NE version 7, Vitek 2 version 8 (bioMerieux, Marcy l'Etoile, France); and RapidID NH System (Thermo Fisher Scientific, Adelaide, Australia), following the manufacturers' instructions.

MALDI-TOF analysis was by bioMerieux MALDI-TOF v2.0 on all three isolates at the laboratory where the initial isolations occurred. For Isolates 2 and 3, Bruker MALDI Biotyper Compass IVD Version 3 (Bruker Daltonik, Bremen, Germany) testing of both isolates was in parallel with known ATCC organisms, in duplicate, to verify the MALDI-TOF and the Vitek system results at genus and species level. These were *Neisseria gonorrhoeae* (ACM 5239), *Moraxella catarrhalis* (ATCC 25238), *Haemophilus influenzae* (ATCC 49766 and NCTC 8468), *Aggregatibacter aphrophilus* (ATCC 7901), *Kingella denitrificans* (ATCC 33394), *Eikenella corrodens* (ATCC BAA115), *Kingella kingae* (ATCC 23330).

Antimicrobial susceptibility testing (AST) was performed according to CLSI guidelines by disc method using Mueller Hinton agar (MHA) with 5% blood defibrinated horse blood (bioMerieux), with incubation in 5% carbon dioxide at 35°C for 24–48 hours, as per the CLSI AST methods for bacterial isolates from animals (2013, 2015) [21, 22] and humans [23–25]. Observed growth on this agar was poor, however the isolates did not grow on any other AST agar. There was no growth on MHA, without blood. As there are no veterinary guidelines for this organism in animals or horses, we used the CLSI guidelines for *Actinobacillus* or *Enterobacteriales* [21, 22]. Beta-lactamase testing was tested using Penicillin (1 Unit) antibiotic discs (Oxoid, Australia), Remel™ Nitrocefin discs (Thermo Fisher, Adelaide, Australia), BD BBL™ Cefinase™ discs for the detection of *Neisseria gonorrhoeae, Staphylocooccus species, Haemophilus influenzae, Moraxella catarrhalis, Enterococci* and anaerobic bacteria for the production of *β*-lactamase. We also used an in-house TGA classified Class 1 IVD Beta-lactamase culture method, an adaptation of the “Gots Test” [26]. It is a Mueller Hinton II agar plate (BBL) containing penicillin and a suspension of *S. aureus* (1-2 McFarland standard). Plates are seeded with a penicillin-sensitive *S. aureus* (ATCC 25992, penicillin MIC = 0.025 mg/L) and containing a concentration of penicillin just above the MIC (0.028 mg/L) of seeded culture prepared in-house. Strains producing *β*-lactamase, when replicated on to the plates, will render the penicillin inactive and allow growth of the seeded strain as a zone around the inoculum. Any organism inhibited by ≤0.028 mg/L penicillin will not grow on the plates. Quality control organisms used for the “Gots Test” were *Staphylococcus aureus* ATCC 29213 (growth indicating positive *β*-lactamase production) and ATCC 25993 (no growth indicating negative *β*-lactamase production).

For scanning electron microscopy (SEM), cells from broth were washed twice in PBS, vortexed, and added to poly-l-lysine coated glass coverslips and left to attach for 30 minutes at room temperature. This was followed by fixation in 5% glutaraldehyde in PBS overnight at 4°C. The glutaraldehyde was removed and the coverslips were washed 5 times in PBS, followed by dehydration in a graded ethanol series at 20%, 30%, 50%, 70%, 80%, 90%, and 95%, for 15 minutes each. This was followed by two 20 minutes exchanges in 100% ethanol, one 20-minute exchange in 2 : 1 ethanol: Hexamethyldisilazane (HMDS), one 20-minute exchange in 1 : 2 ethanol: HMDS, and two 20 minutes exchanges in 100% HMDS. After the final exchange, the samples were left to dry completely in a desiccation chamber for at least 48 hours. Samples were mounted onto specimen stubs, coated in ~5 nm gold/palladium and imaged on a Zeiss Supra 55VP Field Emission Scanning Electron Microscope at an accelerating voltage of 5 kV.

16S rRNA gene profiling was initially performed on Isolate 1, which could not be propagated but material was available from the initial culture. Therefore, a segment of the 16S rRNA gene was amplified using the two-fold degenerate forward primer 27f-CM (F 5′-AGAGTTTGATCMTGGCTCAG, where M is A or C) and the reverse primer 1492r (R 5′-TACCTTGTTACGACTT), described by Frank et al. [27].

*Isolates 2* and *3* could be propagated on subculture. DNA was extracted from a pure culture of the isolates. A total of 2 ml of liquid culture of each isolate was centrifuged and DNA extracted using a combination of bead-beating (5.5 ms^−1^ for 30 s) and the Qiagen AllPrep kit (Qiagen, CA, USA). Two sets of primers were used to amplify segments of two domains of the 16S rRNA. Set A consisted of a universal 1020 base pair (bp) 16S rRNA bacterial primers (Domain III): F 5′-GAC TCC TAC GGG AGG CAG CAG-3′ and R 5′-CTG ATC CGC GAT TAC TAG CGA TTC-3′ [28]. Set B consisted of 490 bp partial sequence of the 16S rRNA gene (Domain I): F 5′-CCT AAC ACA TGC AAG TCG ARCG-3′ and R 5′-CGT ATT ACC GCG GCT GCT-3′ [29]. These were used to amplify this gene using 10 ng of genomic DNA isolated from each strain. 16S rRNA phylogeny based classification was carried out by comparison of the highly conserved 16S rRNA which is part of a small subunit ubiquitously present in the ribosomes of all prokaryotes. Samples were extracted using Qiagen semi-automated BioRobot® (M48) system (Qiagen, CA, USA). Once amplification was complete, PCR products were quantified via electrophoresis (Biorad) and visualized on a Sigma 4% agarose gel stained with ethidium bromide using 1× TBE (0.089 M Tris borate, pH 8.3 containing 2 mN EDTA) buffer system against a molecular ruler. Gel loading buffer contains 0.25% bromophenol blue, 0.25% xylene cyanol, and 40% sucrose. Ethidium bromide (0.5 *µ*g/mL) was included in the gel for easy visualisation of bands under the UV light and to confirm the presence of a 1020 base pair (bp) and 490 bp band. The QIAquick PCR Purification Kit Protocol was used to purify single or double stranded DNA fragments from PCR and other enzymatic reactions. Fragments ranging from 490 to 1020 bp are purified from primers, nucleotides, polymerases and salts preparing the amplified product for sequencing. Sanger sequences were generated using the Bigdye terminator sequencer ABI Science PRISM/Hitachi (Model 3135) Prism Genetic Analyzer (Applied Biosystems, Life Technologies Corporation, Foster City, Foster City, California, USA). Sanger sequences of Isolates 2 and 3, were then analysed on the NCBI GenBank (http://www.ncbi.nlm.nih.gov/entrez/) against other 16S rRNA reference databases for identification. Evolutionary analysis was conducted in MEGA X [30], using the maximum composite likelihood approach of Tamura and Nei [31]. Identical results were obtained for isolates 2 and 3 (Supplementary [Supplementary-material supplementary-material-1]).

Genomic average nucleotide identities were calculated with JSpecies using the ANIb method [32, 33]. Genome-wide average nucleotide identities (gANI), alignment fractions (AF) and intra-species probabilities were calculated using protein-coding genes as described by Varghese et al. [34]. DNA-DNA hybridisation was estimated in silico using the identities/HSP length formula from the Genome-to-Genome Distance Calculator [33].

## 4. Results

Positive culture for all three cases was only obtained from CMM. Time to culture positivity was 4-5 days (see Supplementary [Supplementary-material supplementary-material-1]).

Here we report the features of *Isolate 1* (from Case 1) separately, as it was not available for more detailed testing because it failed to survive repeat subculture. Gram stain morphology of Isolate 1 was Gram negative cocco-bacilli (GNCB) appearing in short, pairs and in long chains. Colonial morphology was small, grey to clear colonies, showing beta-hemolysis on sheep blood agar. API NH Version 3 (bioMerieux, France) yielded positive reactions for glucose, fructose, saccharose and alkaline phosphatase (Code 3120) and an identification of *Haemophilus paragallinarum (Hp)*. Vitek testing was not performed on Isolate 1, as it could not be propagated on repeated subculture. bioMerieux MALDI-TOF v2.0 yielded an identification of Isolate 1 of *Kingella kingae* at a confidence level of 99.9%.

Gram stain morphology of *Isolates 2* and *3* was identical being Gram negative cocco-bacilli (GNCB) appearing in short, pairs and in long chains, but without the typical morphological features of *Kingella, Simonsiella*, and *Alysiella* (Supplementary [Supplementary-material supplementary-material-1]). Individual cells of the equine isolates are wide (6.9–7.3 *µ*m), medium length (1.0–1.2 *µ*m), and relatively oval shaped and attach to form filaments that are 8–12 or more cells long. The long chains had similarities to the characteristic chains seen with *Simonsiella* and *Alysiella* species, as well as occasionally seen with some Moraxella species but were unlike the typical Gram stain appearance of *Kingella kingae* [13].

Colonial morphology of *Isolates 2* and *3* was identical being small, grey to clear colonies, showing beta-hemolysis on sheep and supplemented Columbia horse blood agar [16]. Plates had a distinct “bleach” odour similar to other organisms in the HACEK group. At 24 hours incubation, colonies were smooth in appearance and demonstrated mild pitting of agar, then developed spreading zones (“fried egg” appearance) at 48 hours of incubation (Supplementary [Supplementary-material supplementary-material-1]). Optimal growth was at 37 ± 2°C, with 5% CO_2_ in aerobic conditions, but there was also growth, though less profuse, under anaerobic conditions with 10–15% carbon dioxide enriched atmospheres. There was no growth on MacConkey agar (Supplementary [Supplementary-material supplementary-material-1]). Both Isolates 2 and 3 demonstrated twitching motility.

Both *Isolates 2* and *3* were oxidase positive, with negative results for catalase, superoxol (3%, 10%, and 30%) and nitrate reduction to nitrite. For both Isolates 2 and 3 results for proprietary tests were: ID32 STAPH version 3 (ArgA and PAL positive), API STAPH version 5 (Glucose, Fructose and PAL positive), rapid ID 32 STREP version 4 (ADH, PAL, RIB, SAC positive), API 20 STREP version 8 (PAL & LAP positive); API 20NE version 7 and RapidID NH System both yielded a positive reaction for glucose and oxidase only. For both Isolates 2 and 3, Vitek 2 system (bioMerieux, Marcy l'Etoile, France) yielded low discriminative identifications (“contraindicating biopatterns”). The organisms tested positive to ArgA and LeuA. Identifications such as *Methylobacterium* species, Neisseria cinerea (ProA 99%) and *Moraxella (Neisseria) ovis* (NO_3_ 99%, PheA 92% and ArgA 1%) were also of low discrimination.

Supplementary [Supplementary-material supplementary-material-1] shows selected phenotypic results for *Isolates 2* and *3* (identical results) compared to those of other Gram negative organisms, including *Kingella kingae*.

MALDI-TOF identification by different equipment showed discordant results. bioMerieux MALDI-TOF v2.0 yielded an identification of *Isolates 2* and *3* of *Kingella kingae* at a confidence level of 99.9%. Results from Bruker MALDI-TOF for both Isolates 2 and 3 were *Klebsiella aerogenes* (Score 1.62) followed by *Kingella kingae* as the next highest rank score (1.55), on thrice repeated testing.

For SEM, from Supplementary [Supplementary-material supplementary-material-1], it can be seen that the *Isolates 2* and *3* differ from the *Kingella kingae* control (*Kingella kingae*, ATCC 23330), being more coccoid than rod shaped. Protruding outer membrane vesicles (OMVs) are seen clearly. Images did not resemble published SEM images of *Simonsiella* or *Alysiella* [13].

Antimicrobial susceptibility test results are provided in Supplementary [Supplementary-material supplementary-material-1]. Notably, one isolate was beta-lactamase positive (Isolate 2). *Isolate 2*, tested positive for *β*-lactamase using Remel™ Nitrocefin and Cefinase™ discs as well as the “Gots Test” method described in the methods. *Isolate 1*, tested negative on all the above mentioned *β*-lactamase methods.

16S rRNA analysis, for *Isolate 1* (M12-17630), using the primers as in Frank et al. [27] as described above in Methods, despite excellent resolution at genus level (100% homology), could not separate between *Kingella kingae* and *Kingella denitrificans*, and some divergence (96% homology) was noted between the Isolate 1 and these *Kingella* species. Genbank accession number for this isolate is KR494280 (see Supplementary [Supplementary-material supplementary-material-1]).

16S rRNA analysis, for *Isolates 1, 2,* and *3*, using primers as per references [28, 29], generated the phylogenetic tree provided in Supplementary [Supplementary-material supplementary-material-1]. Isolates 1, 2, and 3 appear to be identical.

Whole genome comparisons between *Isolates 2* and *3* and the type strains of close phylogenetic relatives *Alysiella crassa* and *Kingella kingae* using the ANIb method revealed average nucleotide identities of 76.60 and 76.44%, respectively [35]. These values are well below the generally accepted species threshold of 95-96% ANI [36]. Further analysis using the genome-wide Average Nucleotide Identity (ANI) method produced maximum gANI/AF values of 77.77%/0.54 for *Alysiella crassa* and 77.61%/0.52 for *Kingella kingae*. In both comparisons this equates to a probability of <1.0e-06 that the novel equine isolates belong to either of these species [34]. Finally, calculation of Genome-to-Genome Distance produced in silico DNA-DNA hybridisation (dDDH) values well below the >70% threshold used to demarcate species [35]. Comparison of isolate 3 to *Alysiella crassa* and *Kingella kingae* genomes revealed dDDH values of 25.3% ± 2.4% and 23.4% ± 2.4%, respectively. Again these values equate to a very low probability (≤0.01%) that experimental DNA-DNA hybridisation comparisons would be above the 70% species threshold.

## 5. Discussion

We describe isolation of a novel bacterium from three young thoroughbred horses from different regions in NSW, Australia. Gram stain morphology was Gram negative cocco-bacilli (GNCB) appearing in pairs and in chains, but without the typical morphological features of *Kingella, Simonsiella*, and *Alysiella*.

Phenotypic tests provided conflicting results. Our isolates exhibited unique characteristics but most resembled *Kingella species*, rather than *Simonsiella*, although our isolates showed negative results for catalase and superoxol (3%, 10%, and 30%), nitrate reduction to nitrite (*Simonsiella* and *Alysiella* are both positive for catalase, *Alysiella* is nitrate/nitrite negative), maltose (*Simonsiella* and *Kingella kingae* are positive), and positive results for alkaline phosphatase (*Simonsiella* is negative, *Kingella kingae* is positive). Like our equine isolates, *Kingella kingae* is also nonmotile, and exhibits positive oxidase activity, negative catalase reaction, and produces acid from glucose and maltose but not from other sugars, see Supplementary [Supplementary-material supplementary-material-1].

MALDI-TOF by different equipment also gave conflicting results. Identification of the bacterium was *Kingella kingae* by bioMerieux MALDI-TOF to a level of 99.9% for all 3 isolates. However, using Bruker MALDI Biotyper Compass IVD Version 3 on Isolates 2 and 3, no reliable identification was obtained with best match score values of 1.55.

Based on 16S rRNA analysis, phylogenetic trees showed *Isolates 1, 2,* and *3* to be most closely related to *Kingella kingae* but the average nuclear identity (ANI) was less than 89%. With further 16S rRNA gene sequence analysis, and construction of a phylogenetic tree using isolates of human and of animal origin by MEGA-X neighbouring joining method using the Kimura correlation and bootstrap values calculated from 1000 trees, the Isolates 2 and 3 did not align with any of the *Neisseriaceae species* or nearby neighbours of *Simonsiella muelleri, Alysiella crassa* or *Kingella kingae* (Supplementary Figures [Supplementary-material supplementary-material-1] and [Supplementary-material supplementary-material-1]).

Using a specific PCR for diagnosis of OAI in humans, *Kingella kingae* was found to be the most common cause of septic arthritis in children aged between 6 months and 5 years of age, causing 53% of all 83 cases with proven OAI [37]. In equines, despite efforts to improve the detection of bacterial pathogens in septic arthritis, optimising culture and using 16S rRNA real-time PCR, in almost 60% of cases, no pathogen is identified [38]. In that study, SF was collected into sterile serum collection tubes and trypticase soy broth (TSB). Blood agar plates were directly inoculated and incubated at 35°C for 48 hours (both aerobically and anaerobically). For enrichment, TSB was incubated in aerobic conditions at 35°C for 72 hours then sub-cultured onto blood agar after 24, 48, 72 hours for further incubation. Of 38 samples, 18 (38%) were culture positive. One (1) sample yielded mixed growth. Of the 18 positive cultures, 7 (39%) were obtained from the enrichment cultures. Bacteria were isolated from 7/28 (25%) SF samples from 21 horses known to have received antimicrobial therapy prior to sample collection compared to 5/13 (38%) that had not received antimicrobials. The two (2) most commons organisms cultured from SF samples were *Actinobacillus equuli* (6 isolates) and *Staphylococcus aureus* (5), with the remainder comprised of coagulase negative *Staphylococcus* sp. (2), and one (1) each of the following: *Salmonella species, Enterobacter cloacae, Enterobacter agglomerans, Enterococcus* sp., *Pseudomonas aeruginosa, Streptococcus zooepidemicus*. RT-PCR failed to detect 3/18 (17%) culture positives, but was positive in 4/30 (13%) culture negative samples. The authors did note, however, that some amplicons generated were not in their reference database.

There are no data on epidemiology, microbiology, clinical manifestations or management of *Kingella* infection in horses. The only other animal isolate of *Kingella* is *Kingella potus* isolated from the wound of a zookeeper bitten by a kinkajou (*Potus flavus*) [39]. Taxonomically, *Kingella* species fall within the order *Neisseriales* and the emended family *Neisseriaceae*, which includes the genera *Alysiella, Bergeriella, Conchiformibius, Eikenella, Kingella, Neisseria, Simonsiella, Stenoxybacter, Uruburuella* and *Vitreoscilla, Neisseria* being the type genus of the family [40]. All species are obligate host-associated organisms apart from *Vitreoscilla*, which may be free-living. Whilst the Gram stain appearance of most members of this family is coccal, coccoid, or rod-shaped, as single cells, pairs, masses or short chains, *Alysiella* and *Simonsiella* may form chains of eight (8) cells or more. Flagellae are absent but gliding or twitching motility may be present [40]. Gram stain appearance of our isolates (Supplementary [Supplementary-material supplementary-material-1]) more closely resembles some Moraxella species rather than *Kingella, Alysiella* or *Simonsiella*, the latter two having characteristic morphology.

A novel species isolated from the oral cavity of sheep formed long chains of Gram negative cocci and, although initially classified as *Alysiella* sp., was later reclassified as a new species of *Moraxella* (*Moraxella oblonga*), based on 16S rRNA gene sequence, fatty acid composition, quinone system analysis and biochemical tests [41]. Although SEM appearances are similar, neither biochemical tests (Supplementary [Supplementary-material supplementary-material-1]) nor 16S rRNA gene sequence analysis (Supplementary Figures [Supplementary-material supplementary-material-1] and [Supplementary-material supplementary-material-1]) suggests relatedness of our isolates to *Moraxella* including *Moraxella oblonga*.

*Simonsiella* species are normal oral flora of a diverse range of warm-blooded vertebrates but *Simonsiella* species have only been isolated from a limited number of animals: humans (*S. muelleri*), cats (*Simonsiella sp.*), dogs (*S. steedae*), guinea pigs, rabbits and sheep (*S. crassa*) [42]. Only three (3) species however have been validly described (*S. muelleri, S. steedae*, and *S. crassa*) such that Bergey's Manual of Systematics of Archaea and Bacteria advises that “*Alysiella* has been reported from the oral cavity of many animals but the organism has only been isolated from the mouths of cows, guinea pigs and sheep, with merely a few isolates from sheep having been described in detail” [13]. The genus *Alysiella* originally contained only one species isolated from the mouth of sheep (*Alysiella filiformis*) but the previously named *Simonsiella crassa* (from sheep) has been reclassified to the genus as *Alysiella crassa* based on 16S rRNA sequencing [43]. Whilst there is no report of isolation of *Simonsiella* or *Alysiella* species from equines, almost one hundred years ago, Simons reported the appearance of *Simonsiella*-like organisms on microscopy of oral samples from equines [44]. *Simonsiella* and *Alysiella* are regarded as nonpathogenic in immunocompetent hosts [42]. Our isolates do not show the characteristic morphology of *Alysiella* or *Simonsiella*.

Members of the order *Neisseriales* (which contains a single family – *Neisseriaceae*) have no specific distinguishing biochemical, phenotypic or molecular characteristics. Members may be best distinguished by comparison of conserved signature indels (CSIs) in a number of widely distributed proteins specific for *Neisseriaceae* [40]. Clade 1 contains the genera *Eikenella, Kingella, Neisseria*, and *Simonsiella* – obligate host-associated organisms that lack flagellae and show varied morphology [40].

All horses appeared to make a full recovery with surgical intervention and antibiotic therapy. What effect the infection had on racetrack performance is difficult to determine as there are many variables involved in such performance. In children, OAI caused by *Kingella kingae* may follow a relatively benign course, often resolving without antibiotic therapy or any intervention at all [45].

Further analysis is required to determine the classification of this potentially new species. Until more isolates and clinical case material becomes available, guidelines for AST should follow published veterinary CLSI guidelines, as there is currently inadequate information on AST for equine isolates available from alternative veterinary sources.

The only antibiotics to which all three isolates were tested and found to be susceptible were ceftiofur and tetracycline. Resistance to co-trimoxazole was demonstrated in Isolate 1, and to penicillin, ampicillin and gentamicin in Isolate 2. In fact, Case 2 received only one parenteral antibiotic to which Isolate 2 was susceptible (ceftriaxone given by regional limb perfusion). Two isolates were tested to fluoroquinolones and found to be susceptible. Although all cases appeared to make a full recovery, empiric antibiotic therapy in the treatment of OAI in horses suspected to be caused by this organism should include either a third generation cephalosporin, a tetracycline, or a fluoroquinolone, until further data becomes available.

The epidemiological significance of infection with this organism remains to be determined, but its occurrence in young horses may suggest epidemiological similarities to *Kingella kingae* infections in humans.

## Supplementary Materials

Supplementary Materials Table 1: clinical and laboratory features of three cases of osteoarticular infection caused by a likely novel species. Table 2: phenotypic and biochemical characteristics of the new isolate(s) (2 & 3) compared with Kingella kingae, Moraxella oblonga, Moraxella bovis, and Alysiella filiformis. Figure 1a: gram stain of novel equine bacterial isolate. Figure 1b: gram stain of novel equine bacterial isolate. Figure 2a: novel equine bacterial isolate with beta haemolytic, grey translucent colonies displayed on Horse Blood agar [16] after 24 hours incubation at 36°C in 5% 28 CO _2_. Figure 2b: novel equine bacterial isolate with “fried egg” appearance of colonies displayed on Columbia Horse Blood agar [16] after 72 hours incubation at 36°C. Figure 3a: scanning electron microscopy (SEM) of novel equine bacterial isolate, compared with Kingella kingae. Novel equine isolate is more coccoid than rod-shaped. Protruding outer membrane vesicles (OMVs) are clearly seen. Figure 3b: novel equine bacterial isolate in chains. Figure 3c: novel equine bacterial isolate dimensions (1143 nm × 732.8 nm). Figure 3d: novel equine bacterial isolate dimensions (1143 nm × 732.8 nm). Figure 4: 16S rRNA analysis, for Isolates 1 [27]; Molecular Phylogenetic tree by Maximum Likelihood Bootstrap method outlines the evolutionary history based on Tamura-Nei model [31, 40]. Figure 5: 16S rRNA analysis, for Isolates 2 and 3 [28, 29]; Molecular Phylogenetic tree by Maximum Likelihood Bootstrap method outlines the evolutionary history based on Tamura-Nei model [31, 40].
