# Supplementary material for: Osteoarticular Infection in Three Young Thoroughbred Horses Caused by a Novel Gram Negative Cocco-Bacillus
Source: Case Rep Vet Med. 2020 Jan 18;2020:9785861. doi: 10.1155/2020/9785861 (PMC6994210; doi:10.1155/2020/9785861)
Supplement: Supplementary Materials — Table 1: clinical and laboratory features of three cases of osteoarticular infection caused by a likely novel species. Table 2: phenotypic and biochemical characteristics of the new isolate(s) (2 & 3) compared with Kingella kingae, Moraxella oblonga, Moraxella bovis, and Alysiella filiformis. Figure 1a: gram stain of novel equine bacterial isolate. Figure 1b: gram stain of novel equine bacterial isolate. Figure 2a: novel equine bacterial isolate with beta haemolytic, grey translucent colonies displayed on Horse Blood agar [16] after 24 hours incubation at 36°C in 5% 28 CO 2. Figure 2b: novel equine bacterial isolate with “fried egg” appearance of colonies displayed on Columbia Horse Blood agar [16] after 72 hours incubation at 36°C. Figure 3a: scanning electron microscopy (SEM) of novel equine bacterial isolate, compared with Kingella kingae. Novel equine isolate is more coccoid than rod-shaped. Protruding outer membrane vesicles (OMVs) are clearly seen. Figure 3b: novel equine bacterial isolate in chains. Figure 3c: novel equine bacterial isolate dimensions (1143 nm × 732.8 nm). Figure 3d: novel equine bacterial isolate dimensions (1143 nm × 732.8 nm). Figure 4: 16S rRNA analysis, for Isolates 1 [27]; Molecular Phylogenetic tree by Maximum Likelihood Bootstrap method outlines the evolutionary history based on Tamura-Nei model [31, 40]. Figure 5: 16S rRNA analysis, for Isolates 2 and 3 [28, 29]; Molecular Phylogenetic tree by Maximum Likelihood Bootstrap method outlines the evolutionary history based on Tamura-Nei model [31, 40]. [file 9785861.f1.pdf]

Osteoarticular infection in three young thoroughbred horses caused by a novel Gram negative cocco-bacillus

Supplementary Tables & Figures

Table 1: Clinical and laboratory features of three cases of osteoarticular infection caused by a likely novel species (see text for further details, comment and any abbreviations). *Number of relevant reference in "References".

Characteristic	Case 1	Case 2	Case 3
**Clinical**			
Age	15 months	7 months	3 months
Sex	Female	Female	Male
Duration of Symptoms (days)	14	> 3	Not Stated
Lameness (Degree)	Yes (4/5)	Yes (4/5)	Yes (2/5)
Fever (Centigrade)	Yes (38.9)	No	No
Systemically Unwell	No	No	No
Tenosynovitis	Yes	Yes	Yes
Arthritis	Yes	No	No
Osteomyelitis	Yes	Yes	No
Site	Right bicipital bursa	Foreleg (third metacarpal bone - McIII)	Hind digital flexor tendon sheath
**Imaging**			

Characteristic	Case 1	Case 2	Case 3
Plain X-rays	Normal	Abnormal: defect in superficial cortex of McIII	Normal
Ultrasound	Large volume echoic mass in bicipital bursa, likely fibrinous material	Confirmed bone defect seen on x-ray	Effusion hind digital flexor tendon sheath
**Treatment**			
Surgical Intervention	Yes (lavage, bone debridement)	Yes (lavage, bone debridement)	Yes (lavage)
Antibiotic Treatment (Days) Intravenous Unless Stated Otherwise (RLP = regional limb perfusion)	Enrofloxacin (5)<br>Benzyl penicillin (5)<br>Oxytetracycline (NS)	Ceftriaxone (RLP)<br>Amikacin beads<br>Gentamicin (NS)<br>Procaine penicillin (NS)<br>Sulfadimidine/trimethoprim (7)	Gentamicin (8)<br>Benzyl penicillin (8)<br>Sulfadimidine/trimethoprim (5)
Outcome at 6 weeks	Fully recovered	Fully recovered	Fully recovered
Racetrack Performance	Winner	No Data	Unplaced
**Laboratory Tests**			

Characteristic	Case 1	Case 2	Case 3
**Bloods**			
Complete blood count abnormalities	Hemoglobin 121g/L	Nil	Not Stated
Fibrinogen (Normal Range: 2-4g/L)	7g/L	5g/L	Not Stated
**Synovial Fluid**			
Protein (Normal: < 25g/L)	38g/L	Not Stated	47g/L
Cell Count (x109/L)	0.3	Not Stated	257 (96% neutrophils)
**Histopathology**			
Tissue - Abnormality	Synovial biopsy bicipital bursa - subacute largely purulent proliferative synovitis; no organisms seen	Nil (bone submitted for microbiology only)	Nil (synovial only sent for analysis)
**Microbiology**			
Day culture became positive from Cooked Meat Medium (CMM)	Day 5	Day 5	Day 4

Characteristic	Case 1	Case 2	Case 3
Sample Positive	Fibrin from lavage	Bone from debridement	Fibrin from lavage
MALDI-TOF Identification (Bruker & bioMerieux 2.0)	*Kingella kingae* *bioMerieux 99.9%* *Bruker score 1.55*	*Kingella kingae* *bioMerieux 99.9%* *Bruker score 1.55*	*Kingella kingae* *bioMerieux 99.9%* *Bruker score 1.55*
Antibiotic Susceptibilities of novel isolates [33,34,35, 40, 41, 42] EUCAST/CLSI methods*[35, 40, 41, 42,47]	**Susceptible*** to: penicillin (PEN1), ceftiofur (EFT30), tetracycline (TE30), ciprofloxacin (CIP5). **Resistant** to: co-trimoxazole (SXT25) . Nil other agents tested.	**Susceptible*** to: ceftriaxone (CRO30), ceftiofur (EFT30), tetracycline (TE30), co-trimoxazole (SXT25), enrofloxacin. **Resistant** to: penicillin (PEN1), ampicillin (AMP10), gentamicin (CN10), neomycin (N30UI) (Beta-lactamase positive by BD BBL Cefinase Test & Class 1 in-house IVD "Gots Test" a β-lactamase culture method adapted from Gots J.S. [48])	**Susceptible*** to all agents tested: penicillin (PEN1), gentamicin (CN10), ceftiofur (EFT30), tetracycline (TE30), chloramphenicol (C30), amikacin (AMC3). Nil other agents tested.

* EUCAST/CLSI Disc Diffusion Method was used for confirmatory and comparative susceptibility analyses on control and isolate subcultures using the fastidious organisms guidelines and relevant referenced breakpoints [35,40,41, 42, 47]

Table 2: Phenotypic and biochemical characteristics of the new isolate(s) (2 & 3) compared with *Kingella kingae*, *Moraxella oblonga*, *Moraxella bovis*, *Alysiella filiformis*.

**Characteristic**	**New isolates (2 & 3)**	***K.kingae****	***M.oblonga****	***M.bovis****	***A.filiformis****
**Morphology**	Cocci, chains	Rods	Cocci, chains	Rods	Filamentous
**Beta-haemolysis on blood agar**	+	+	+	+	+
**Catalase**	-	-	-	+	+
**Oxidase**	+	+	+	+	+
**Nitrate reduction**	-	-	-	+	-
**Acid production from:**					
Glucose	+[a,d]	+	-	-	+
Maltose	-	+	+	-	-
**Hydrolysis of:**					
Arginine	+[c,f]	-	+	+	-
Gelatin	-	-	+	+	-

Lysine	**NT**	**-**	**-**	**+**	**-**
Ornithine	**-**	**-**	**-**	**-**	**-**
Urea	**-**	**-**	**-**	**-**	**-**
Alkaline Phosphatase (PAL)	**+**[a,b,c]				
**Indole production**	**-**	**-**	**-**	**-**	**-**

+ = positive reaction; - = negative reaction; NT = Not tested; *Data sourced from references [12, 13, 29, 30, 31].

[a] API STAPH v5.0; [b]Rapid ID 32 STREP v4.0; [c]ID 32 STAPH v3.0; [d]RapidNH v8.0; [f]VITEK 2 v8.0.

Figure 1a. Gram stain of novel equine bacterial isolate

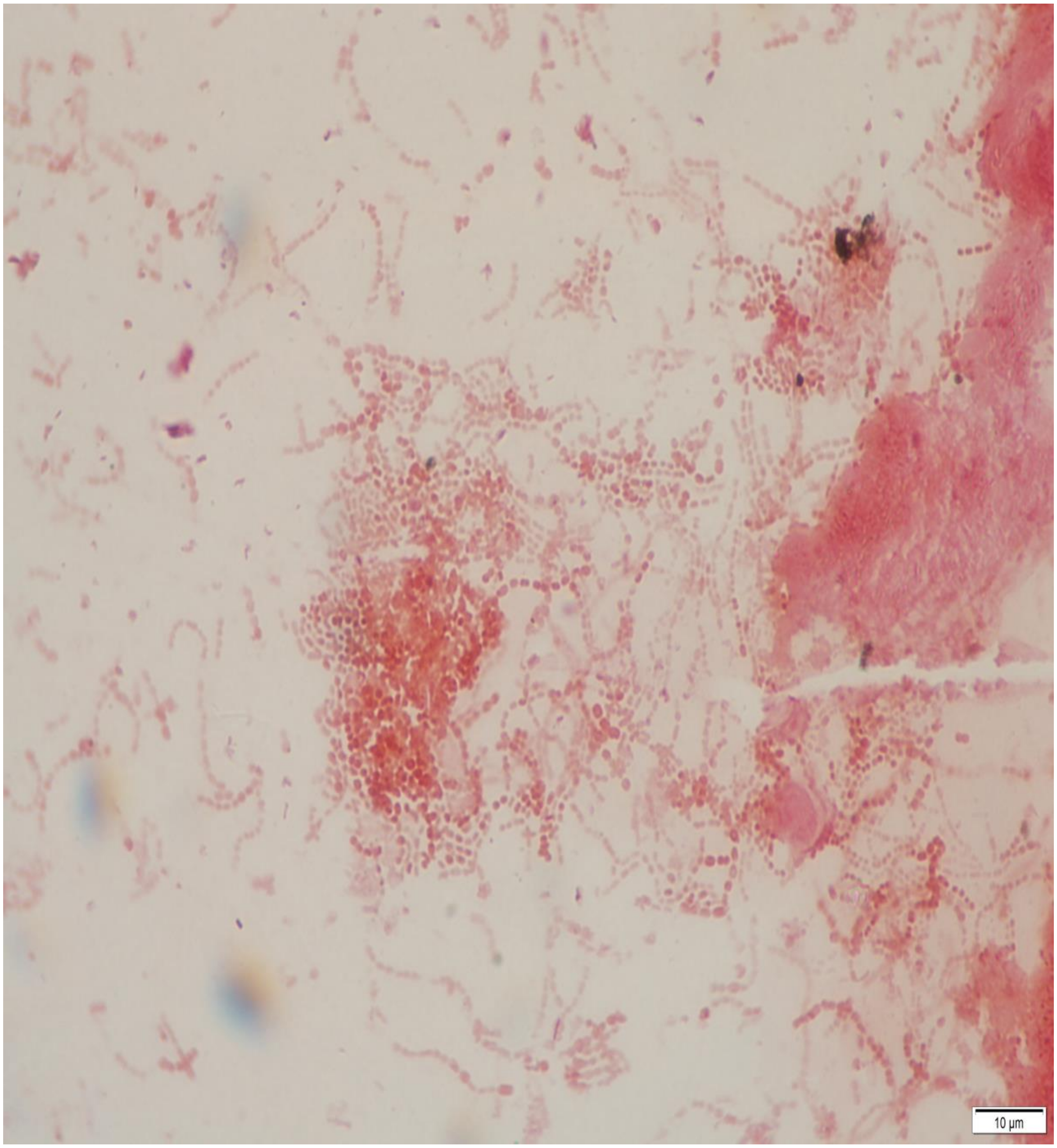

Figure 1b. Gram stain of novel equine bacterial isolate

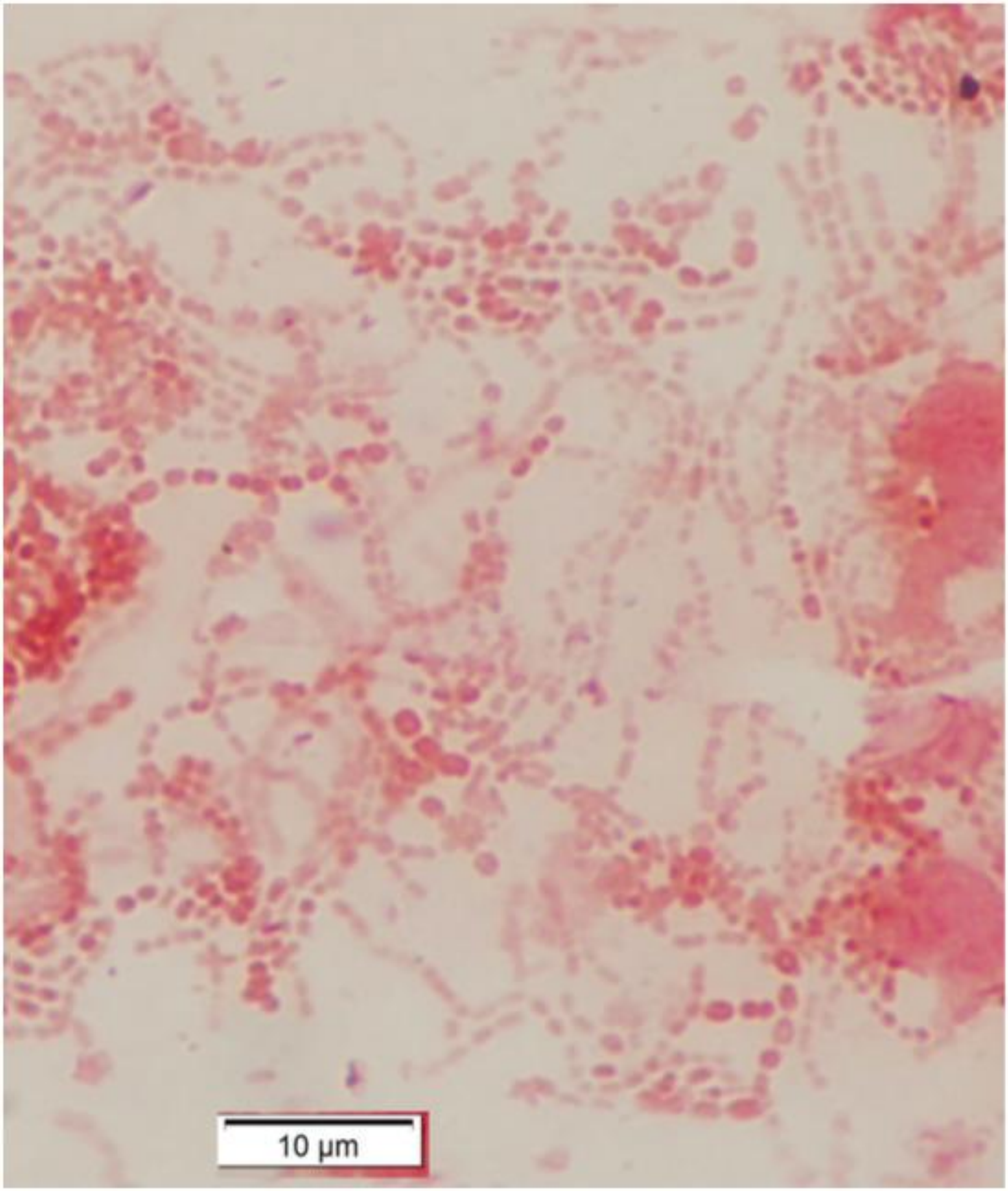

Figure 2a. Novel equine bacterial isolate with beta haemolytic, grey translucent colonies displayed on Horse Blood agar [46] after 24 hours incubation at 36◦C in 5% CO_2.

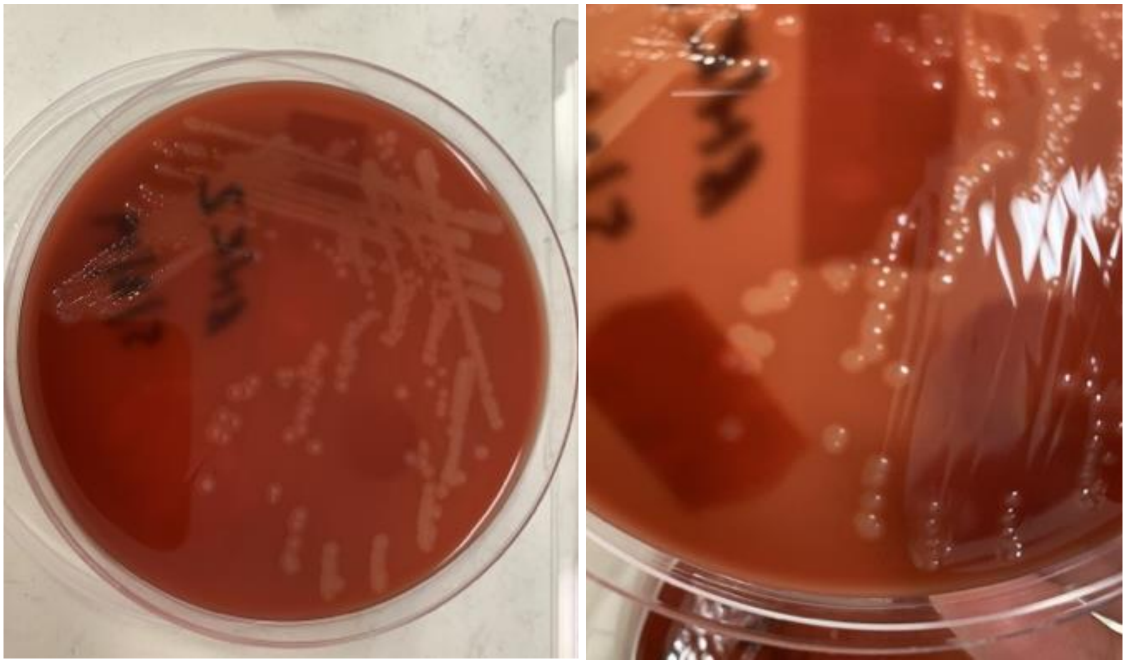

Figure 2b. Novel equine bacterial isolate with “fried egg” appearance of colonies displayed on Columbia Horse Blood agar [46] after 72 hours incubation at 36◦C.

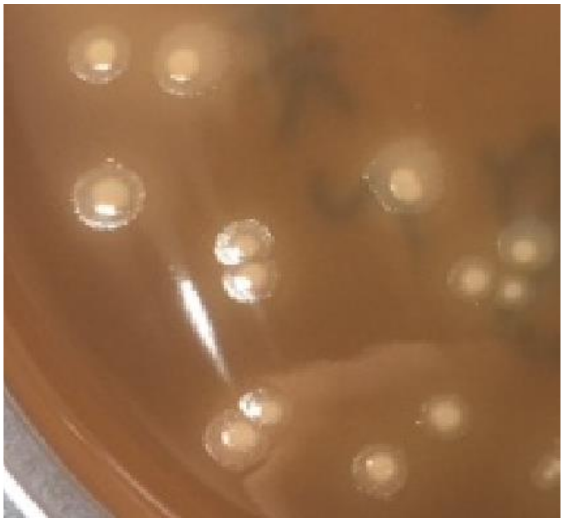

Figure 3a: Scanning electron microscopy (SEM) of novel equine bacterial isolate, compared with *Kingella kingae*. Novel equine isolate is more coccoid than rod-shaped. Protruding outer membrane vesicles (OMVs) are clearly seen.

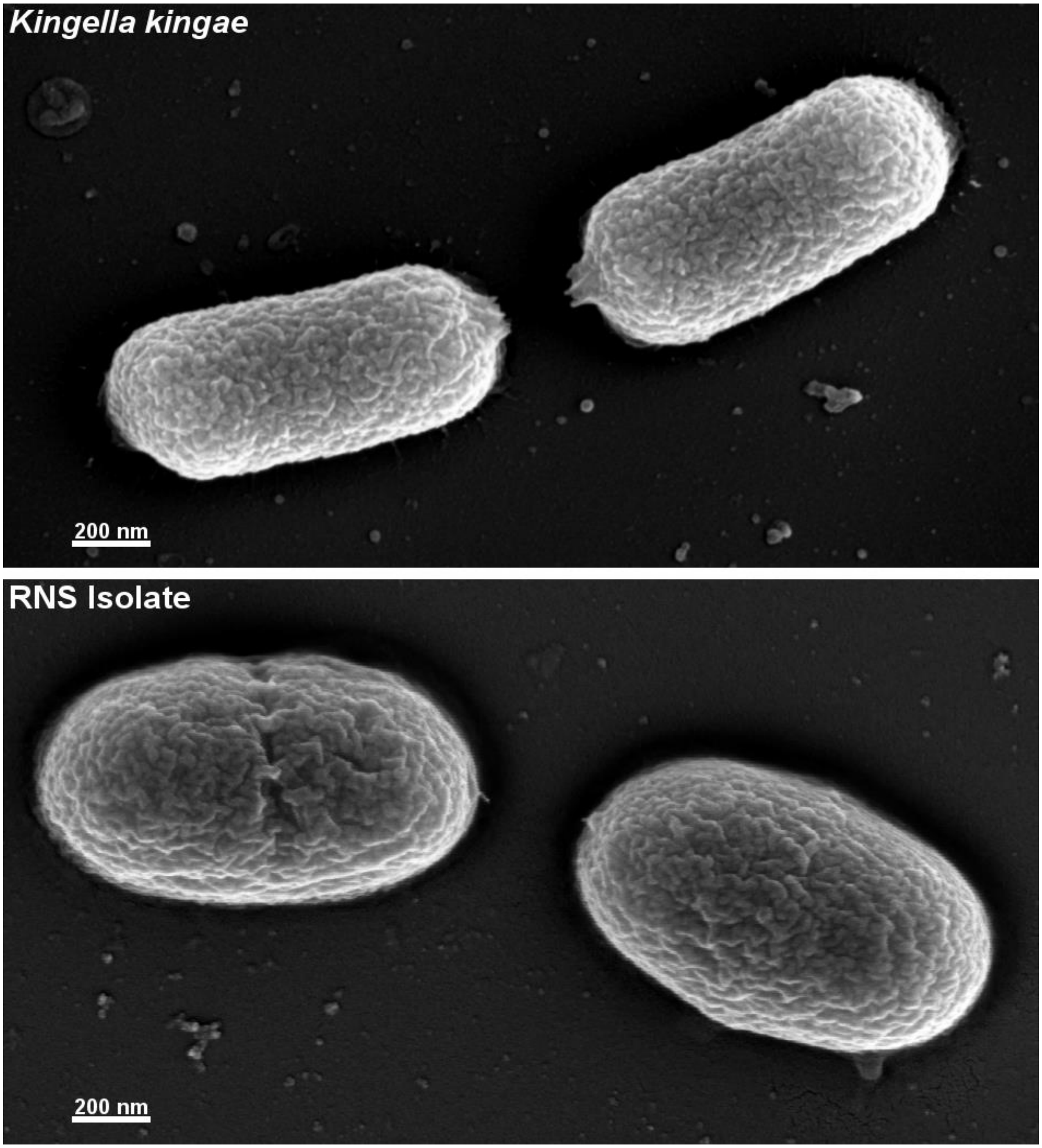

Figure 3b. Novel equine bacterial isolate in chains

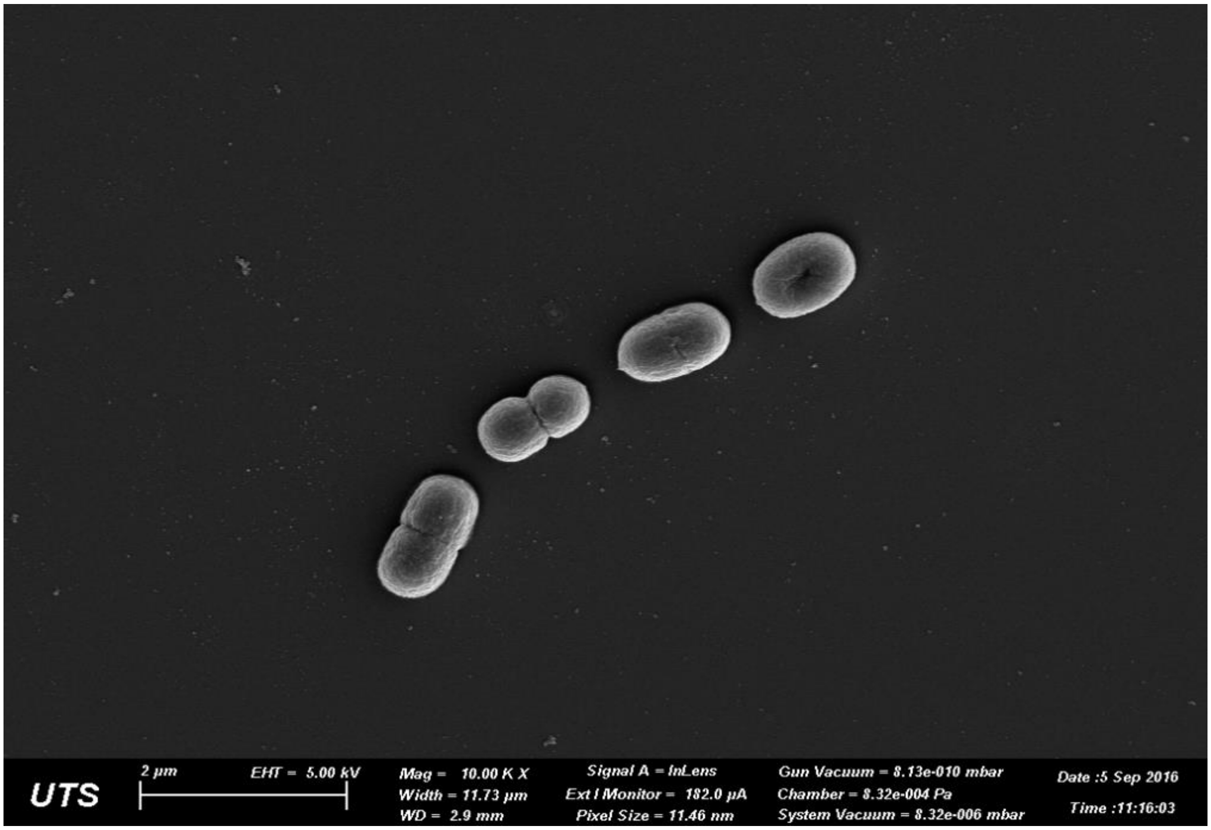

Figure 3c. Novel equine bacterial isolate dimensions (1143nm x 732.8nm)

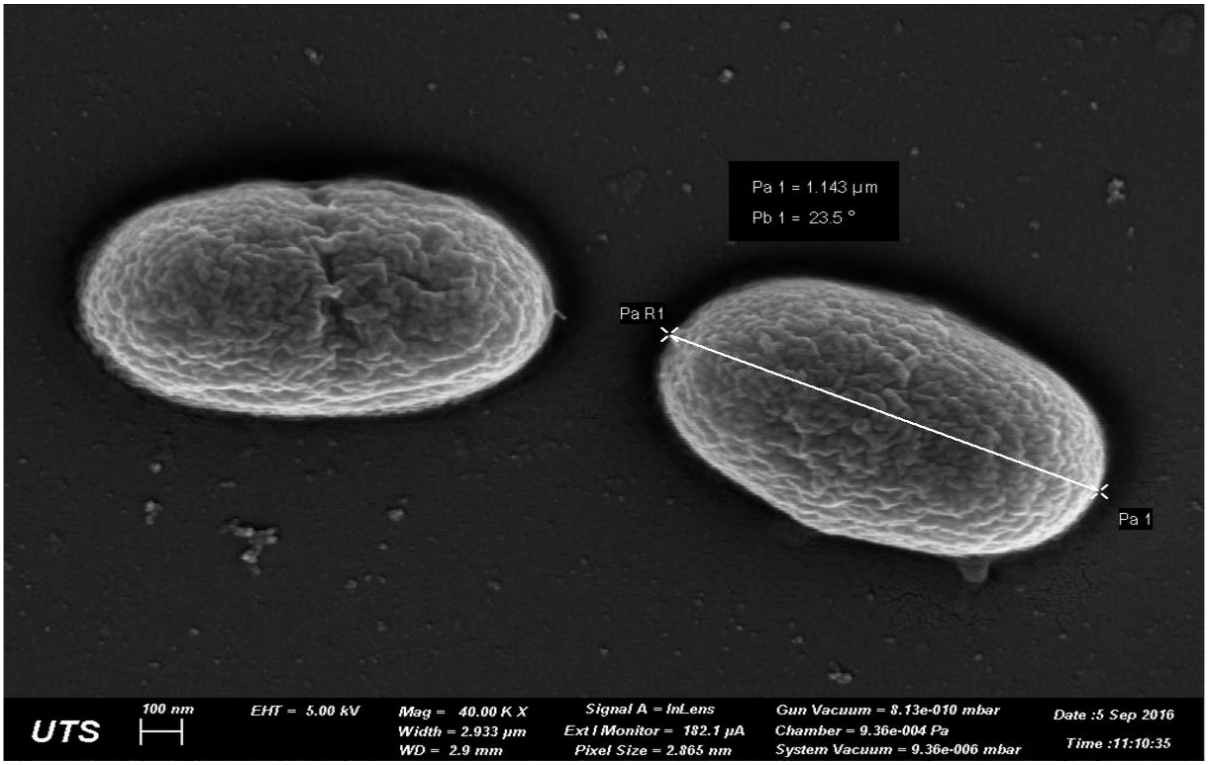

Figure 3d. Novel equine bacterial isolate dimensions (1143nm x 732.8nm)

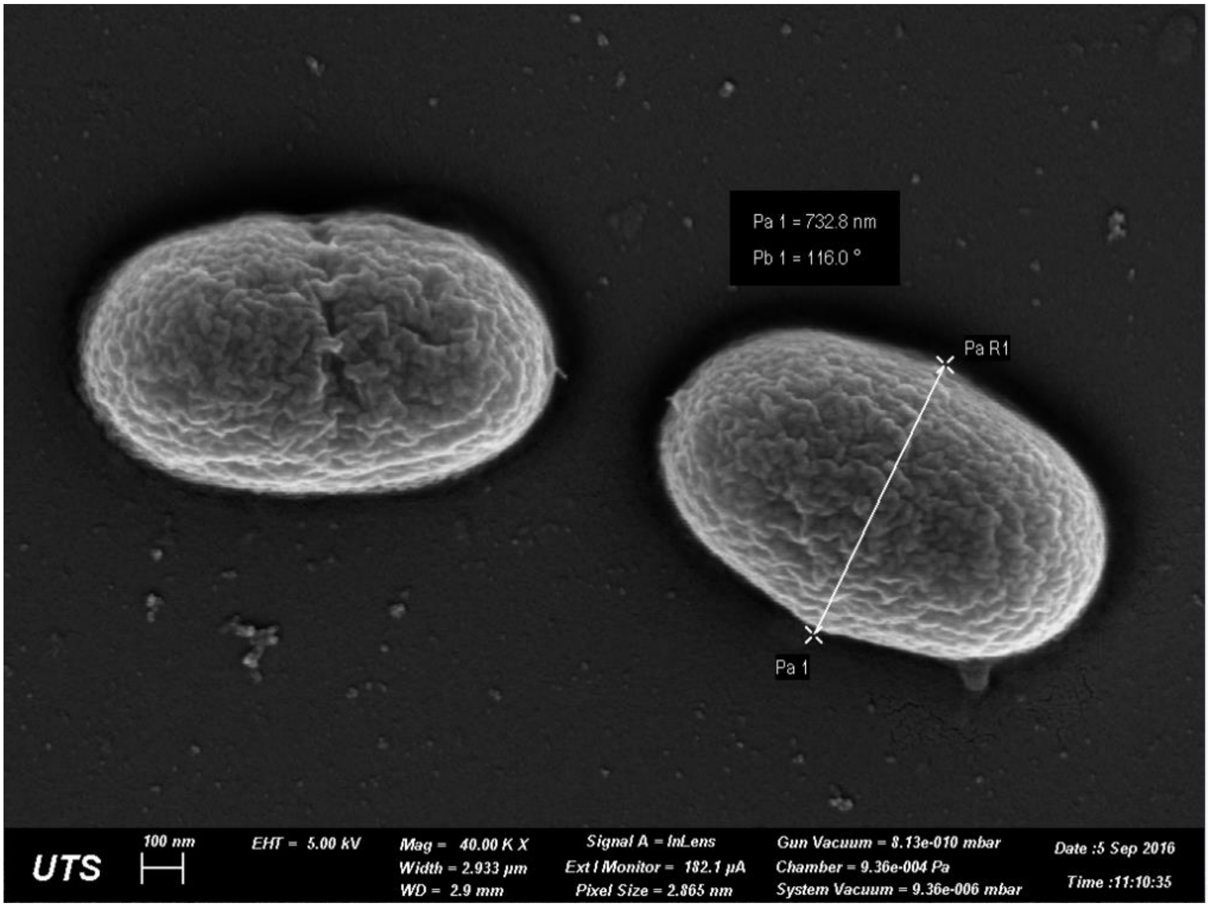
Pa 1 = 732.8 nm
Pb 1 = 116.0 °
Pa R1
Pa 1
UTS
100 nm
EHT = 5.00 kV
Mag = 40.00 K X
Width = 2.933 µm
WD = 2.9 mm
Signal A = InLens
Ext I Monitor = 182.1 µA
Pixel Size = 2.865 nm
Gun Vacuum = 8.13e-010 mbar
Chamber = 9.36e-004 Pa
System Vacuum = 9.36e-006 mbar
Date :5 Sep 2016
Time :11:10:35

Figure 4: 16S rRNA analysis, for **Isolates 1** [16]; Molecular Phylogenetic tree by Maximum Likelihood Bootstrap method outlines the evolutionary history based on Tamura-Nei model [18, 27]

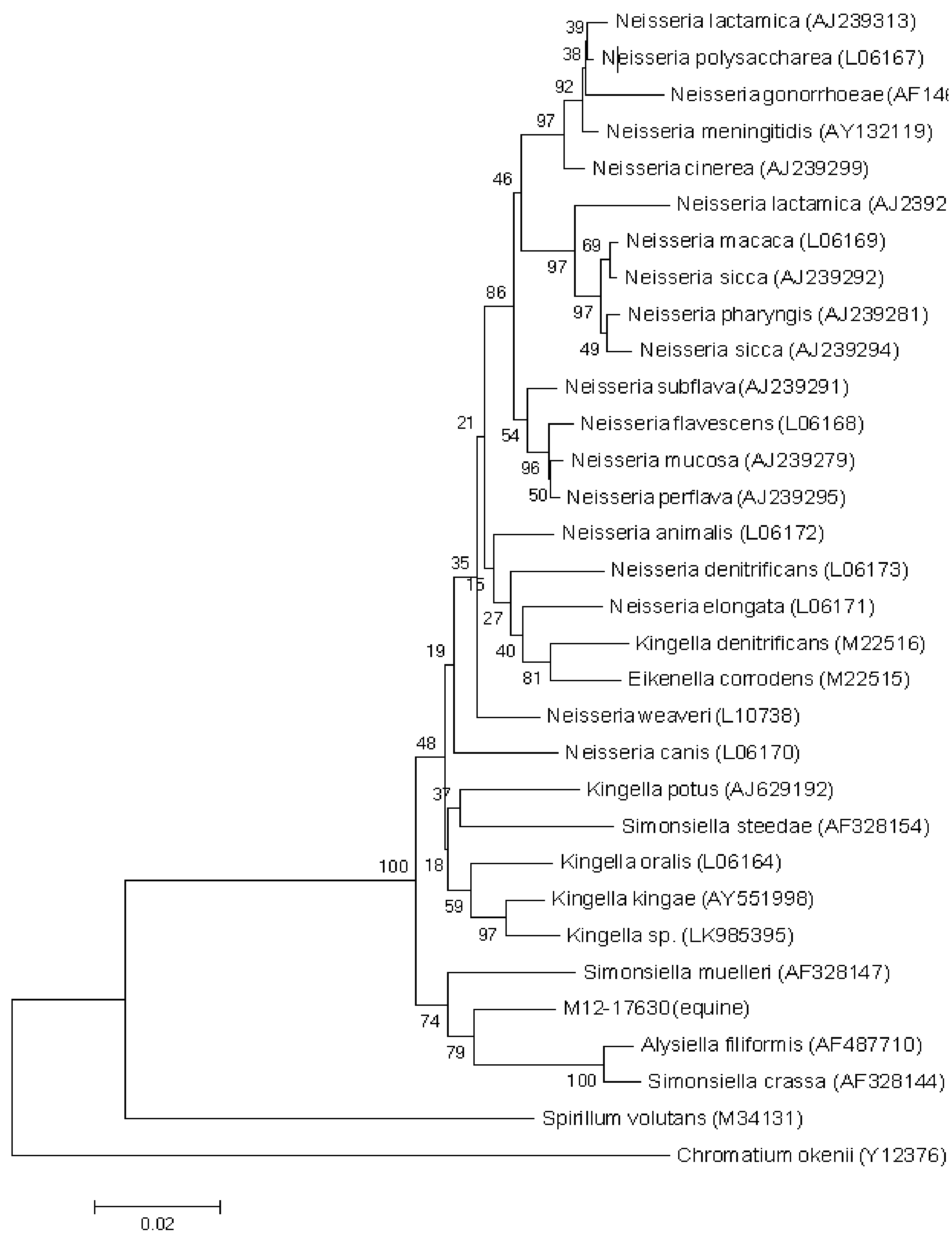

Figure 5: 16S rRNA analysis, for **Isolates 2** and **3** [44, 45]; Molecular Phylogenetic tree by Maximum Likelihood Bootstrap method outlines the evolutionary history based on Tamura-Nei model [18, 27]

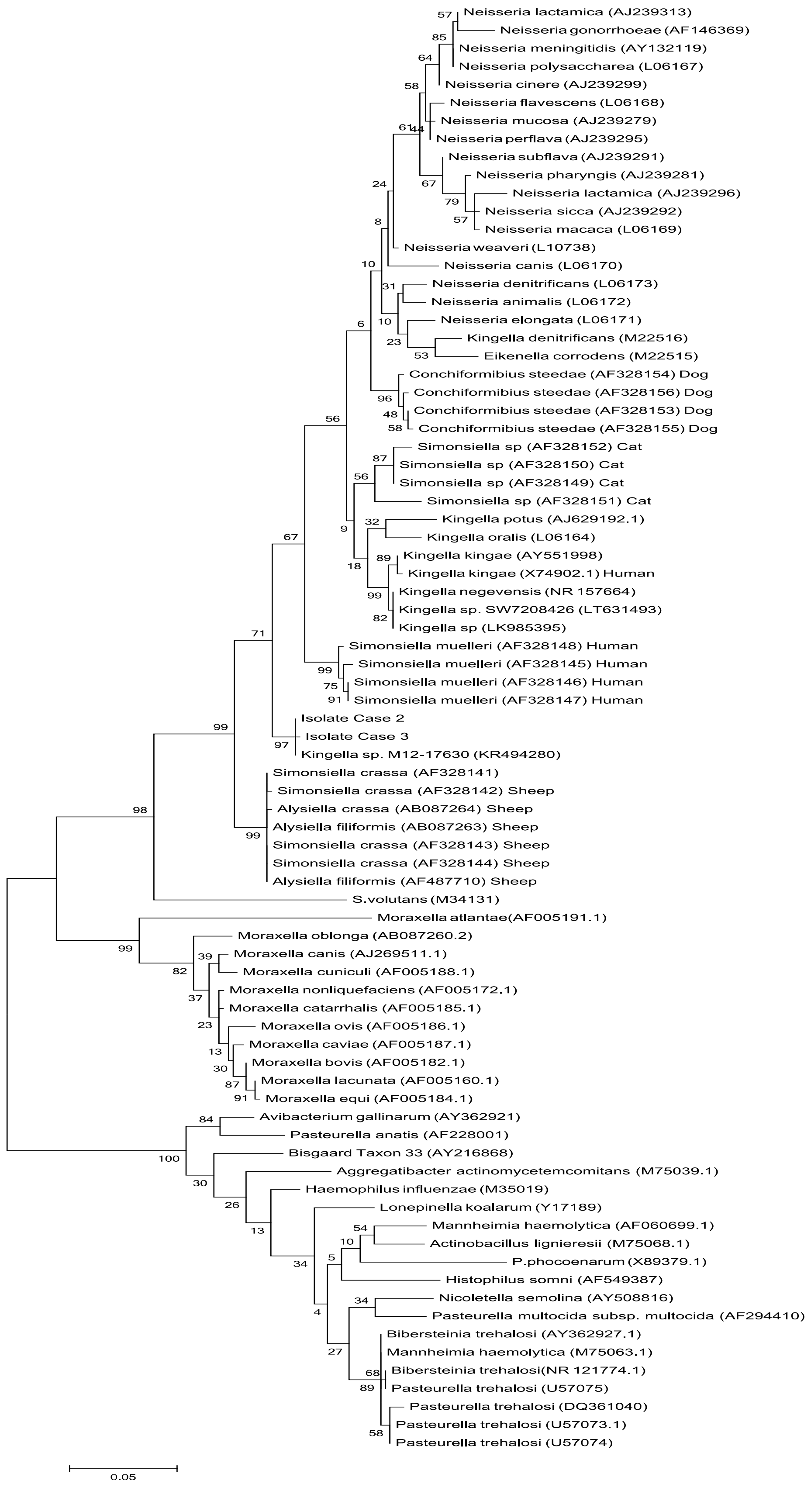

Neisseria lactamica (AJ239313)
Neisseria gonorrhoeae (AF146369)
Neisseria meningitidis (AY132119)
Neisseria polysaccharea (L06167)
Neisseria cinere (AJ239299)
Neisseria flavescens (L06168)
Neisseria mucosa (AJ239279)
Neisseria perflava (AJ239295)
Neisseria subflava (AJ239291)
Neisseria pharyngis (AJ239281)
Neisseria lactamica (AJ239296)
Neisseria sicca (AJ239292)
Neisseria macaca (L06169)
Neisseria weaveri (L10738)
Neisseria canis (L06170)
Neisseria denitrificans (L06173)
Neisseria animalis (L06172)
Neisseria elongata (L06171)
Kingella denitrificans (M22516)
Eikenella corrodens (M22515)
Conchiformibius steedae (AF328154) Dog
Conchiformibius steedae (AF328156) Dog
Conchiformibius steedae (AF328153) Dog
Conchiformibius steedae (AF328155) Dog
Simonsiella sp (AF328152) Cat
Simonsiella sp (AF328150) Cat
Simonsiella sp (AF328149) Cat
Simonsiella sp (AF328151) Cat
Kingella potus (AJ629192.1)
Kingella oralis (L06164)
Kingella kingae (AY551998)
Kingella kingae (X74902.1) Human
Kingella negevensis (NR 157664)
Kingella sp. SW7208426 (LT631493)
Kingella sp (LK985395)
Simonsiella muelleri (AF328148) Human
Simonsiella muelleri (AF328145) Human
Simonsiella muelleri (AF328146) Human
Simonsiella muelleri (AF328147) Human
Isolate Case 2
Isolate Case 3
Kingella sp. M12-17630 (KR494280)
Simonsiella crassa (AF328141)
Simonsiella crassa (AF328142) Sheep
Alysiella crassa (AB087264) Sheep
Alysiella filiformis (AB087263) Sheep
Simonsiella crassa (AF328143) Sheep
Simonsiella crassa (AF328144) Sheep
Alysiella filiformis (AF487710) Sheep
S.volutans (M34131)
Moraxella atlantae(AF005191.1)
Moraxella oblonga (AB087260.2)
Moraxella canis (AJ269511.1)
Moraxella cuniculi (AF005188.1)
Moraxella nonliquefaciens (AF005172.1)
Moraxella catarrhalis (AF005185.1)
Moraxella ovis (AF005186.1)
Moraxella caviae (AF005187.1)
Moraxella bovis (AF005182.1)
Moraxella lacunata (AF005160.1)
Moraxella equi (AF005184.1)
Avibacterium gallinarum (AY362921)
Pasteurella anatis (AF228001)
Bisgaard Taxon 33 (AY216868)
Aggregatibacter actinomycetemcomitans (M75039.1)
Haemophilus influenzae (M35019)
Lonepinella koalarum (Y17189)
Mannheimia haemolytica (AF060699.1)
Actinobacillus lignieresii (M75068.1)
P.phocoenarum (X89379.1)
Histophilus somni (AF549387)
Nicoletella semolina (AY508816)
Pasteurella multocida subsp. multocida (AF294410)
Bibersteinia trehalosi (AY362927.1)
Mannheimia haemolytica (M75063.1)
Bibersteinia trehalosi(NR 121774.1)
Pasteurella trehalosi (U57075)
Pasteurella trehalosi (DQ361040)
Pasteurella trehalosi (U57073.1)
Pasteurella trehalosi (U57074)
0.05
